# Lipid Composition of *Sheffersomyces stipitis* M12 Strain Grown on Glycerol as a Carbon Source

**DOI:** 10.17113/ftb.58.02.20.6540

**Published:** 2020-06

**Authors:** Stela Križanović, Damir Stanzer, Jasna Mrvčić, Karla Hanousek-Čiča, Elizabeta Kralj, Gordana Čanadi Jurešić

**Affiliations:** 1 Division for Marine and Environmental Research, Ruđer Bošković Institute, Bijenička 54, 10000 Zagreb, Croatia; 2 Department of Food Engineering, Faculty of Food Technology and Biotechnology, University of Zagreb, Pierottijeva 6, 10000 Zagreb, Croatia; 3 Karlovac University of Applied Science, Trg Josipa Jurja Strossmayera 9, 47000 Karlovac, Croatia; 4 Department of Medical Chemistry, Biochemistry and Clinical Chemistry, Faculty of Medicine, University of Rijeka, B. Branchetta 20, 51000 Rijeka, Croatia

**Keywords:** lipid composition, *Sheffersomyces stipitis*, *S*-adenosyl-l-methionine production, lipid metabolism scheme

## Abstract

**Research background:**

In this study the content and composition of lipids in ergosterol-reduced *Sheffersomyces stipitis* M12 strain grown on glycerol as a carbon source is determined. Blocking the ergosterol synthesis route in yeast cells is a recently proposed method for increasing *S*-adenosyl-l-methionine (SAM) production.

**Experimental approach:**

The batch cultivation of M12 yeast was carried out under aerobic conditions in a laboratory bioreactor with glycerol as carbon source, and with pulsed addition of methionine. Glycerol and SAM content were monitored by high-performance liquid chromatography, while fatty acid composition of different lipid classes, separated by solid phase extraction, was determined by gas chromatography.

**Results and conclusion:**

Despite the reduced amount of ergosterol in yeast cells, thanks to the reorganized lipid metabolism, M12 strain achieved high biomass yield and SAM production. Neutral lipids prevailed (making more than 75% of total lipids), but their content and composition differed significantly in the two tested types of yeast. Unsaturated and C18 fatty acids prevailed in both the M12 strain and wild type. In all fractions except free fatty acids, the index of unsaturation in M12 strain was lower than in the wild strain. Our tested strain adjusts itself by changing the content of lipids (mainly phospholipids, sterols and sterol esters), and with desaturation adjustments, to maintain proper functioning and fulfil increased energy needs.

**Novelty and scientific contribution:**

Reorganization of *S. stipitis* lipid composition caused by blocking the metabolic pathway of ergosterol synthesis was presented. A simple scheme of actual lipid metabolism during active SAM production in *S. stipitis,* grown on glycerol was constructed and shown. This fundamental knowledge of lipid metabolic pathways will be a helpful tool in improving *S. stipitis* as an expression host and a model organism, opening new perspectives for its applied research.

## INTRODUCTION

*S*-adenosyl-l-methionine, also known as SAM, is a molecule essential for the human organism, because it is involved in numerous important biochemical pathways. It is the main methyl donor in methylation reactions in living cells, participating in the biosynthesis of choline, carnitine, creatine and methylation of DNA and proteins. Disruption of SAM metabolism is associated with various disorders and, because of that, it is often used as a food supplement. Considering the broad spectrum of its application (it has been clinically applied in the treatment of liver disease, depression, fibromyalgia, osteoarthritis, and Alzheimer’s disease) and the obtained results, it is important to discover efficient and low-cost ways for its production (*1*, *2*). SAM is formed in yeast cells from d,l-methionine and adenosine triphosphate (ATP). Stimulating SAM synthesis, which consumes both d,l-methionine and ATP, activates a metabolic switch that led to enhanced stress resistance and longevity (*3*). Yeasts are a group of microorganisms capable of producing various valuable metabolites, including SAM. They, unlike other microorganisms, have the ability to accumulate large amounts of SAM during growth on the substrate rich in d,l-methionine (*4*). In the process of microbial SAM production different strategies are applied, such as optimizing growth conditions and/or applying mutagenic reagents or genetic engineering methods to develop more productive yeast strains (*1*, *2*). The intracellular concentration of SAM can be improved by transforming yeast cells with the ethionine-resistance gene (*5*), producing nystatin-resistant mutants with insufficient ergosterol biosynthesis (*6*), as well as by a disruption of the adenosine kinase (ADO1) gene in the methionine salvage pathway (*7*).

In our previous work (*8*) we investigated the production of SAM using yeast *Sheffersomyces stipitis* CBS 5776 (previously *Pichia stipitis*). To increase SAM content in the yeast biomass, UV mutagenesis and nystatin selection were applied. Proteome analyses of the obtained *S. stipitis* M12 strain showed that the disruption of C-24 methylation in ergosterol biosynthesis, a step mediated by C-24 sterol methyltransferase (Erg6p), results in greater SAM accumulation, but increases sensitivity to ethanol, compared to the wild strain (*8*). Yeast *S. stipitis* M12 produced the highest amount of SAM in the early stationary phase on the O-medium to which 6 g/L d,l-methionine were added (*9*). Likewise, the supplementation of glycerol (3% *V/V*) into the O-medium at high process aeration influenced the physiological characteristics of M12 strain (*10*). In this case, *S. stipitis* M12 can simultaneously use glucose and glycerol as sources of carbon and energy. M12 strain first uses glucose, and after exhausting it, it uses glycerol, resulting in the highest biomass yield (compared with growth on glucose only, or glucose with pulsed addition of d,l-methionine). Moreover, the addition of glycerol results in reduced lag phase, and increased content of SAM in the biomass. The content of ergosterol in M12 strain, lacking Erg6, expectedly decreased (*10*). Increased sensitivity to ethanol (representing stress), as well as to other types of stress is related to cell lipid structural modifications (*11*). Moreover, the ability of cells to alter the degree of unsaturation in their membrane lipids is an important factor in cellular acclimatization to environmental conditions (*12*).

Following our previous research, the aim of this study is to examine the possibility of developing a SAM production process with increased yield using glycerol as a carbon source in *S. stipitis* M12 and wild type yeast strains. The identification of main lipid constituents, their content and composition in M12 strain compared to wild type was also examined. Although Shobayashi *et al.* (*6*) proposed the method of blocking the ergosterol synthesis route for increased SAM production process, so far there has been no description of the effect of this procedure on the other cell lipids. Also, there are papers dealing with yeast *Pichia/Sheffersomyces* and glucose as the primary source of carbon in which the lipid composition is fully characterized (*13*-*15*), but articles with other carbon sources are not so common (*16*). In addition, numerous maps of biochemical pathways for yeast *S. cerevisiae* and *S. pastoris* (*Komagataella phaffii*) on glucose as a source of carbon have been published (*15*-*17*), while maps for other carbon sources are not so frequent. For this reason, a simple scheme of active lipid metabolism and active metabolite production with glycerol as a carbon source in *S. stipitis* wild and M12 strain is also given in this paper. The pathway scheme was constructed on the basis of available data (for *P. pastoris* grown on glucose (*13*, *15*, *18*), on *n*-alkanes (*19*) on glycerol and other carbon sources (*20*-*22*)). This fundamental knowledge of lipid metabolic pathways will be a helpful tool in improving *S. stipitis* as an expression host and a model organism, opening new perspectives for its applied research.

## MATERIALS AND METHODS

### Strains and cultivation media

*Sheffersomyces stipitis* CBS 5776 wild type was obtained from DSMZ (German Collection of Microorganisms and Cell Cultures GmBH, Braunschweig, Germany). A SAM-accumulating strain *S. stipitis*, M12, was obtained by UV irradiation of CBS 5776 strain at a radiant exposure of 160 J/m^2^ and subsequent isolation on YPD medium containing nystatin 15 μg/mL (*8*, *9*). YPD medium contained 1% yeast extract, 2% peptone and 2% glucose (all Biolife, Milan, Italy). The yeast biomass grew in a 5-litre Biostat A laboratory bioreactor (Sartorius, Göttingen, Germany), with 3 L of modified O-medium (5% d-glucose, 1% peptone, 0.6% (NH_4_)_2_SO4, 0.5% yeast extract, 0.4% KH_2_PO_4_, 0.2% K_2_HPO_4_ and 0.05% MgSO_4_·7H_2_O, pH=6) (*23*), where glucose was replaced with 5% (*V/V*) glycerol (Sigma-Aldrich, Merck, Darmstadt, Germany) at 30 °C and pH=6. At the beginning of cultivation, 6 g/L of d,l-methionine (Sigma-Aldrich, Merck) were added into the modified O-medium, and the same concentration of d,l-methionine (6 g/L) was added after the yeast entry in the stationary phase.

### Determination of substrate and yeast metabolites in the fermentation medium

The growth increase of the tested strains was measured and expressed in grams of absolutely dry cell mass per litre. Dry cell mass was measured gravimetrically. After centrifugation (10 min, 2650×*g*; Rotofix 32, Hettich GmbH & Co. KG, Tuttlingen, Germany) of 5 mL of sample (yeast cells harvested during and at the end of the cultivation), the pellet was resuspended in 5 mL of distilled water. After repeated centrifugation, the pellet was dried to the constant mass at 104 °C in a pre-weighed tube. Glycerol and SAM content were monitored by high-performance liquid chromatography (HPLC). SAM was quantified by the method of Valkó *et al.* (*24*). Briefly, cells were collected and washed twice with sterilized distilled water. Cell lysis was done by the addition of 0.25 mL of ethyl acetate and 1 mL of 1 M H_2_SO_4_ to 2 mL of yeast suspension, and shaking for 30 min. After the total volume of the sample was settled to 10 mL with distilled water, it was centrifuged at 4000×*g* for 10 min. SAM was analysed in the supernatant using ChromSep HPLC Column SS (250 mm×4.6 mm), guard column IonoSpher 5C with Varian Prostar 230 (Varian, Palo Alto, CA, USA) pumping system and UV lamp at 260 nm. The mobile phase (flow rate 1 mL/min) was ammonium formate buffer (0.25 mol/L, pH=4.0±0.1)/methanol (90:10; *V/V*). The quantity of SAM was calculated from the peak area based on a standard calibration curve. SAM standard was purchased from Sigma-Aldrich, Merck. Glycerol was measured with ProStar Varian 230 analytical HPLC equipped with Varian Pro Star 350 refractive index detector. For separation, Varian MetaCarb 87H column (300 mm×6.5 mm) was used (*25*). Prior to analysis, the samples were centrifuged in a portable centrifuge (Rotofix 32; Hettich GmbH & Co. KG) at 3500×*g* for 10 min, then Carrez reagents (Merck) were added to the supernatant, and the precipitated proteins were removed by filtration.

### Lipid analysis

Wild type yeast cells were harvested at the 17th and 18th hour of cultivation, while M12 cells were harvested from the 30th to the 33rd hour of cultivation (at the beginning of stationary phase) and converted to spheroplasts by the method of Zinser and Daum (*26*), additionally tested by Čanadi Jurešić and Blagović (*27*) and slightly modified for *Pichia* (*15*). Briefly, cells were sedimented by centrifugation (5 min, 3500×*g*; Rotina 420R; Hettich GmbH & Co. KG) and cellular pellet was incubated for 10 min at 30 °C in 0.1 M tris(hydroxymethyl)aminomethane sulfate (Tris-SO_4_, pH=9.4) containing 10 mM dithiothreitol (Sigma-Aldrich, Merck). After centrifugation, the pellet was washed with 1.2 M sorbitol (Sigma-Aldrich, Merck) and suspended for 1 h at 30 °C in a buffer containing 1.2 M sorbitol, 20 mM potassium phosphate and Zymolyase 20T (Seikagaku Corp.,Tokyo, Japan). In this step, spheroplasts were prepared and after centrifugation used in the next step, the extraction of lipids. Total cell lipids were prepared according to Folch *et al.* (*28*) with chloroform/methanol (2:1, *V/V*) as solvents. All solvents were of analytical grade (Carlo Erba, Milan, Italy). Total cell lipids were separated and purified by solid phase extraction using an aminopropyl-bonded silica gel column (Bond-Elut, 3 mL, 500 mg sorbents; Varian, Palo-Alto, CA, USA), according to the method of Giacometti *et al.* (*29*). The solid-phase extraction cartridges were also from Varian. Total lipids extracted from wild type and M12 cells were dissolved in chloroform (10 mg/mL) and separated to nonpolar lipids (NL), polar lipids (PL) and free (nonesterified) fatty acids (FFA) by the solvent system proposed by Giacometti *et al.* (*29*). The fraction of nonpolar lipids was further separated, using the same type of column and following the same method, to sterol esters (SE), triacylglycerols (TAG) and sterols. Diacylglycerols (DAG) and monoacylglycerols (MAG), as possible fractions, were not detected. All fractions (NL, PL, FFA, SE, TAG and sterols) were quantified gravimetrically.

### Determination of fatty acid composition

Fatty acid (FA) composition of total lipids and all separated fractions (NL, PL, FFA, SE and TAG) was determined by gas chromatography (GC) analysis of the corresponding methyl esters. FAs of all tested lipids were transmethylated with methanol/*n*-hexane/sulphuric acid (75:25:1, *V/V/V*) at 90 °C for 90 min, extracted in petroleum ether and analysed by GC. The GC analyses of fatty acid methyl esters (FAMEs) were carried out using an Auto System XL (Perkin-Elmer, Norwalk, CT, USA) with flame-ionization detector (FID) and a capillary column model SP-2560 (100 m×0.25 mm×0.2 µm; Supelco, Bellefonte, PA, USA). Chromatography software TotalChrom 6.3.2. (*30*) was used for data acquisition. The temperatures of the injector and detector were set at 300 and 350 ºC, respectively. The analyses were carried out in a programmed temperature mode as follows: 1 min isothermally at 140 °C (initial oven temperature), 5 °C/min to 180 °C, 4 °C/min to 240 °C and then isothermally for 15 min. Helium (5.0 grade; Messer, Sulzbach, Germany) was used as the carrier gas. The injected sample volume was 1 µL with split injection (100:1). FAMEs were identified by comparison with the commercial standard mixture (lipid standards: FAMEs mixtures C8:0–C24:0; Sigma-Aldrich, Merck). Analyses were done at least in triplicate and the data were reported as percentage of relative area. The degree of unsaturation was expressed as index of unsaturation (IU) and it represents the sum of the mass fraction of each FA multiplied by the number of double bonds, as follows:

IU=(*w*(monoenes)+2 *w*(dienes)+3 *w*(trienes)+4 *w*(tetraenes))/100 /1/

### Statistical methods

The collected data were statistically evaluated using the data analysis software system Statistica v. 13.0. (*31*). The results are shown as mean value±standard deviation. Differences were tested by Mann-Whitney *U* test, and the level of significance was set at p<0.05.

## RESULTS AND DISCUSSION

### Cell growth and SAM production

**Fig. 1:**
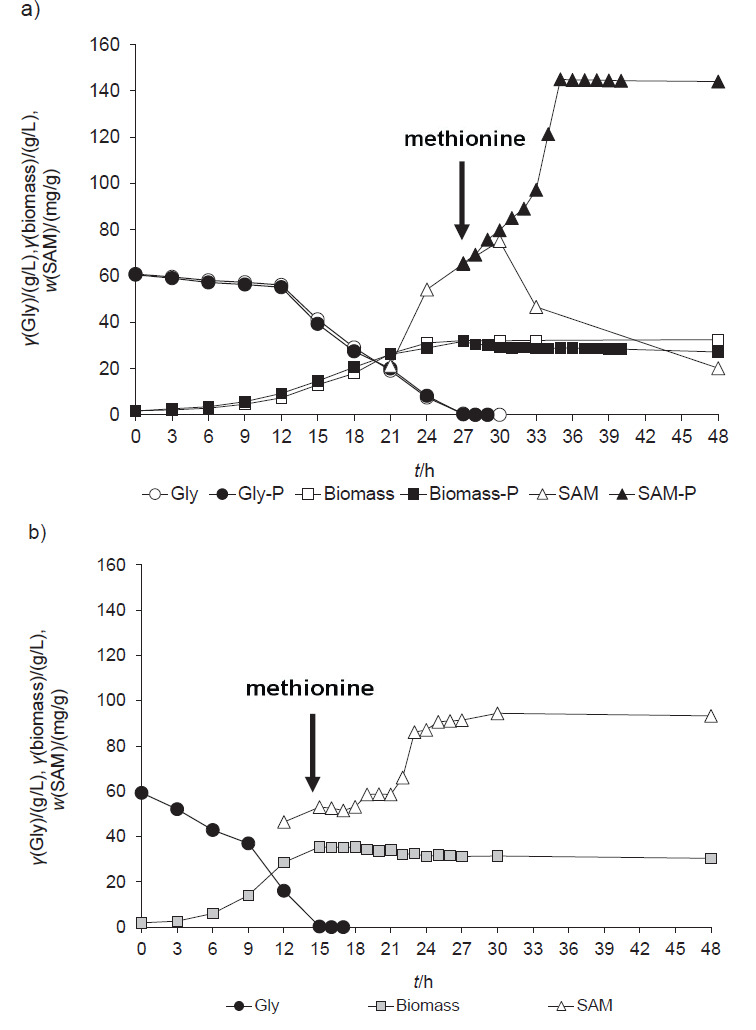
Changes of: a) *Sheffersomyces* stipitis M12 biomass concentration, S-adenosyl-l-methionine (SAM) mass fraction in the M12 biomass, and glycerol (Gly) consumption during cultivation in modified O-medium with 6 g/L d,l-methionine (blank symbols) and pulse supplement 6 g/L d,l-methionine (P) at the 27th hour of cultivation (full symbols) in a laboratory bioreactor (pH=6 and temperature 30 °C), b) *Sheffersomyces stipitis* (wild type) biomass concentration, SAM content in the biomass *Sheffersomyces stipitis* (wild type), and glycerol consumption during cultivation in modified O-medium with 6 g/L d,l-methionine and pulse supplementation of 6 g/L d,l-methionine at the 15th hour of cultivation in a laboratory bioreactor (pH=6 and temperature 30 °C)

Following our previous experiments described above, in this study the batch cultivation of *S. stipitis* M12 yeast was carried out under aerobic conditions in a laboratory bioreactor with glycerol as the main carbon source in the O-medium. In order to achieve better process productivity and higher accumulation of SAM, methionine was added with a pulse at the beginning of the stationary growth phase when the synthesis of SAM is the highest (Fig. 1a).

At the moment of entry into the stationary growth phase (27th hour of growth, Fig. 1a), strain M12 exhausted glycerol and 32.08 g/L of biomass on dry mass basis and a specific growth rate of *μ*=0.11h^-1^, twice as high as the specific growth rate of yeast *S. cerevisiae* on glycerol (*μ*=0.05 h^-1^), were achieved (*32*). During the cultivation, glycerol conversion to biomass (*Y*x/s) of 0.51 g/g was achieved, which corresponds to the *Y*x/s obtained during the growth of wild strain *S. stipitis* on glucose (0.50 g/g) and glycerol (0.56 g/g) (Fig. 1b). The obtained *Y*x/s of M12 strain on glycerol is higher than that obtained during growth of this strain on glucose or a mixture of glycerol and glucose (*Y*x/s=0.17 and 0.32 g/g, respectively, data not shown). /Fig. 1/

These results are consistent with the research of Rumbold *et al.* (*33*) who found that *S. stipitis* CBS 6054 with a 2.0 (mL_air_*/*mL_liquid_)/min aeration rate had the same *Y*x/s during growth on glucose and on glycerol. The results of this part of our research showed that, despite the reduced amount of ergosterol in the cell (*8*) and oxidative stress conditions due to high aeration in the reactor, thanks to the protective role of glycerol, strain M12 can achieve high biomass yield and high SAM production, especially with the pulsed addition of methionine at the beginning of the early stationary growth phase. Using the M12 strain with a pulsed addition of 6 g/L methionine, it was possible to produce on dry mass basis 28.85 g/L of biomass with 145.00 mg/g of SAM in 35 h, while the wild strain *S. stipitis* produced 31.39 g/L of biomass with 94.41 mg/g of SAM. According to literature data, the growth of yeast cells on different carbon sources is most likely associated with the different lipid profile (both the content and composition of lipids) (*15*, *19*).

Therefore, in this research, our aim was to make a comparative profile of lipid content of yeast *S. stipitis* (wild type) and M12 strain grown on glycerol, as well as to find out how the obtained cells reorganize lipid metabolism in response to the disrupted pathway of ergosterol synthesis.

### The content and composition of lipids of S. stipitis (wild type and M12)

#### Total lipid content and composition

Lipids, organic substances soluble in nonpolar organic solvents but not in water, are lately categorized into eight classes: fatty acids (FA), glycerolipids, glycerophospholipids, sterols and sterol derivatives, sphingolipids, prenol lipids, glycolipids, and polyketides (*34*). Besides their importance as components of the cell membranes, they contribute to various processes such as cell signalling, energy supply and cell death. Various organelles such as the endoplasmic reticulum, mitochondria, peroxisomes, and lipid droplets are involved in yeast lipid metabolism (*17*). Yeast cell metabolism fulfils cellular lipid requirements by different pathways (*de novo* synthesis, uptake of external lipids, and turnover of lipids).

Similar to other yeasts, genus *Sheffersomyces/Pichia* balances the synthesis and turnover of (nonpolar) lipids in order to maintain lipid homeostasis. The mass fraction of total lipids on dry mass basis (Table 1) in wild type and M12 strain is low (5.56 and 6.05%, respectively) and in agreement with the data found in literature (*35*). Neutral lipids made the majority of total lipids, and their content and composition differed significantly in the two tested types of yeast. In wild type triacylglycerols (TAG) prevailed (35%) and sterol esters (SE) made up only 14%, while in M12 strain, with impaired sterol metabolism, neutral lipids made up almost 80% of total lipids. In M12 strain TAG also prevailed, the content of SE was high and made up almost 25% while the content of polar lipids changed significantly, compared to wild type, and was lower than sterols (Table 1). Prevalence of TAG over SE is a specific and remarkable feature of *Pichia* genus (*13*, *15*, *36*) and is in strong contrast to *S. cerevisiae*, which contains equivalent amounts of TAG and SE (*37*).

**Table 1:** Total lipids and lipid fractions (extracted from the cells harvested at the beginning of stationary phase) and separated by solid phase extraction in *Sheffersomyces stipitis* wild type and M12 yeast strain grown on glycerol as the main carbon source

(*m*_total lipid_/*m*_dry biomass_)/%	Wild type							
*w*(total lipid)/%							
Neutral lipids					Polar lipids		FFA
5.56±0.03		75.0±1.9				23.7±0.6		1.3±1.8
	SE		TAG		Sterols
	13.9±1.2		35.0±1.4		23.2±2.0
(*m*_total lipid_/*m*_dry biomass_)/%	M12							
	*w*(total lipid)/%							
	Neutral lipids			Polar lipids			FFA	
6.0±0.5	(79.9±2.4)* (15.5±0.9)* (4.6±1.1)*							
	SE		TAG		Sterols
	(24.5±0.9)*		33.0±2.0		(19.5±0.5)*

Results are expressed as mean value±standard deviation (S.D.) and represent the mean value of at least three independent experiments performed in duplicate; *significantly different from the wild type (Mann-Whitney *U* test, p<0.05). SE=steryl esters, TAG=triacylglycerols, FFA=free fatty acids

To avoid accumulation of free sterols and their possible toxicity to the cells, it is very important to balance and regulate the synthesis of sterol esters. Under aerobic conditions, excess of ergosterol can be produced, which may induce several protective pathways. SE synthesis and sequestration in lipid droplets is one of that pathways (*17*). Therefore, sterol biosynthesis and SE formation are connected through a regulatory mechanism. Sterols are necessary for maintaining membrane integrity and they are essential for the viability of eukaryotic cells. It is known that yeast’s main sterol is ergosterol (*17*). In *Pichia* grown on glucose, ergosterol is the main sterol, but there are also present (sorted by quantity): 4,4-dimethylcholesta-8,24-dienol, zymosterol, 4-methylzymosterol, fecosterol, episterol and lanosterol (*15*). In *Pichia* grown on *n*-alkanes, 4-desmethylsterols made 26% of lipids (among them ergosterol prevailed, followed by zymosterol), followed by lanosterol, fecosterol and 4-methylzymosterol (*19*). According to that, the prevalence of ergosterol and all mentioned sterol assortment in wild type of *S. stipitis* is also expected, while in M12 strain, besides an ergosterol and fecosterol, the presence of zymosterol, lanosterol, 4-methylzymosterol, squalene epoxide and squalene is expected. Unlike the wild type, in M12 strain, only sterols that have hydroxyl group at the C3 position of a sterol molecule (like zymosterol, 4-methylzymosterol and lanosterol) can actually participate in the formation of SE, because the biosynthetic pathway is disrupted at the C24 methylation step. The C3 hydroxyl group is necessary for SE formation, so the authors assume that in M12 strain, besides ergosterol and fecosterol, the sterols: zymosterol, lanosterol and some other monomethylsterols, make part of regulatory mechanism and can be transformed to SE. The content of SE in M12 strain, more than 10% greater than in wild type, goes in favour of that theory. In M12 strain, the absence of Erg6p disrupts C24 methylation as one of the important reactions utilizing methionine and results in the accumulation of SAM as methionine donor in this reaction. Thus, during cultivation, besides SAM, M12 strain also accumulates sterol intermediates, and these intermediates are mainly esterified to SE.

Converting the sterols to SE is a way for cells to overcome possible toxicity of sterols (*17*) but FFAs also represent a threat. The content of FFA in M12 strain is significantly higher than in the wild type (Table 1). They are commonly linked to a glycerol backbone to form TAG. The higher content of FFA and lower content of TAG in M12 strain than in the wild type of *S. stipitis* indicates the degradation of TAG.

**Fig. 2:**
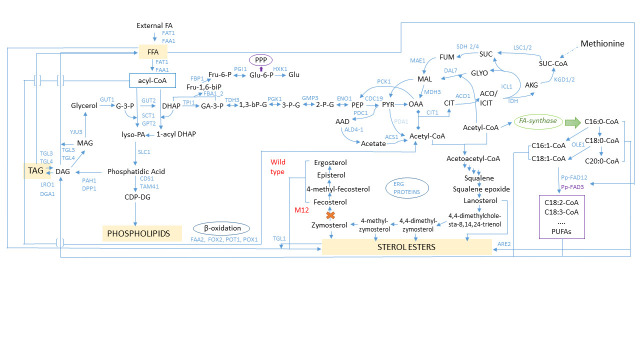
Network of actual biochemical pathways constructed for *S. stipitis* wild and M12 type grown on glycerol. The network is mainly constructed on the base and data analysis given by Prielhofer *et al.* (*20*) for *P. pastoris* grown on excess of glycerol. The data from Ivashov *et al*. (*13*, *18*), Klug (*15*), Tae Myoung *et al.* (19), Wei *et al.* (*21*) and Zhang *et al.* (*22*) were also implemented in this network. **Metabolites:** AAD=acetaldehyde, ACO=aconitate, AKG=α-ketoglutarate, CIT=citrate, DAG=diacylglycerols, 1-acyl DHAP=1-acyl dihydroxyacetone phosphate, DHAP=dihydroxyacetone phosphate, F-1,6-biP=fructose 1,6-bisphosphate, Fru-6-P=fructose 6-phosphate, FFA=free fatty acids, FUM=fumarate, G-3-P=glycerol 3-phosphate, GA-3-P=glyceraldehyde 3-phosphate, Glu-6-P=glucose 6-phosphate, GLYO=glyoxylate, ICIT=isocitrate, lyso-PA=lysophosphatidic acid, MAG=monoacylglycerols, MAL=malate, OAA=oxaloacetate, 1,3-bPG=1,3-bisphosphoglycerate, 2-P-G=2-phosphoglycerate, 3-P-G=3-phosphoglycerate, PEP=phosphoenolpyruvate, PPP=pentose phosphate pathway, PUFA=polyunsaturated fatty acids, PYR=pyruvate, SUC=succinate, SUC-CoA=succinyl-coenzyme A, TAG=triacylglycerols. **Enzymes:** ACO1/2=aconitase, ACS1=acetyl-coA synthetase, ALD4=mitochondrial aldehyde dehydrogenase, ARE2=acyl-CoA:sterol acyltransferase, CDC19=pyruvate kinase, CDS1=phosphatidate cytidylyltransferase, CIT1=citrate synthase, DAL7=malate synthase, DGA1=diacylglycerol acyltransferase, DPP1=diacylglycerol pyrophosphate (DGPP) phosphatase, ENO1=enolase I, ERG PROTEINS=enzymes involved in ergosterol synthesis, FAA1=long chain fatty acyl-CoA synthetase, FAA2=medium chain fatty acyl-CoA synthetase, FAD3=ω-3 desaturase, FAD12=Δ^12^-fatty acid desaturase, FAT1=very long chain fatty acyl-CoA synthetase and long chain fatty acid transportase, FBA1-1/1-2=fructose 1,6-bisphosphate aldolase, FBP1=fructose-1,6-bisphosphatase, FOX2=multifunctional enzyme of the peroxisomal fatty acid β-oxidation pathway (it has 3-hydroxyacyl-CoA dehydrogenase and enoyl-CoA hydratase activities), FUM1=fumarase, GPD1=glycerol-3-phosphate dehydrogenase, GPM1/3=phosphoglycerate mutase, GPT2=glycerol 3-phosphate/dihydroxyacetone phosphate dual substrate-specific sn-1 acyltransferase, GUT1=glycerol kinase, GUT2=glycerol-3-phosphate dehydrogenase, HXK1=hexokinase, ICL1=isocitrate lyase, IDH1/2=isocitrate dehydrogenase, KGD1=α-ketoglutarate dehydrogenase complex, KGD2=dihydrolipoyl transsuccinylase, LSC1=succinyl-CoA ligase, LRO1=diacylglycerol acyltransferase, MAE1=mitochondrial malic enzyme, MDH1=mitochondrial malate dehydrogenase, MDH3=malate dehydrogenase, OLE1=Δ^9^-fatty acid desaturase, PAH1=Mg2+-dependent phosphatidate (PA) phosphatase, PCK1=phosphoenolpyruvate carboxykinase, PDA1=E1 α subunit of the pyruvate dehydrogenase (PDH) complex, PFK1/2=phosphofructokinase, PGI1=phosphoglucose isomerase, PGK1=3-phosphoglycerate kinase, POT1=3-ketoacyl-CoA thiolase, POX1=Fatty-acyl coenzyme A oxidase, PYC2=pyruvate carboxylase, SCT1=glycerol 3-phosphate/dihydroxyacetone phosphate dual substrate-specific *sn*-1 acyltransferase, SDH 2/4=succinate dehydrogenase, SLC1=1-acyl-*sn*-glycerol-3-phosphate acyltransferase, TAM41=mitochondrial phosphatidate cytidylyltransferase (CDP-DAG synthase), TGL1=steryl ester hydrolase, TGL3/4=triacylglycerol lipase, TPI1=triose phosphate isomerase, TDH3=glyceraldehyde-3-phosphate dehydrogenase, YJU3=monoglyceride lipase (MGL)

On the other hand, TAG could be formed by acylation of diacylglycerol (DAG) with FA (Fig. 2). The fraction of DAG was not detected in any of the tested yeast (Table 1), so it can be assumed that this fraction is metabolically very active, and that all synthesised DAG are used immediately for further processes.

Phospholipids made the largest part of polar lipid (PL) fraction. They consist of DAG as a hydrophobic side, glycerol backbone moiety and different polar head groups as hydrophilic side, linked to the sn-3 position of glycerol. In DAG, glycerol backbone is esterified with FA at the sn-1 and sn-2 positions. The lowest content of phospholipids and complete PL fraction in M12 strain, together with the absence of DAG and MAG fractions could indicate the excessive metabolic activity during SAM production.

#### Fatty acid content and composition

Fatty acids can be incorporated into phospholipids and sphingolipids or serve as an energy reservoir in TAG and SE. The FA composition of *S. stipitis* was relatively simple. Major detected FAs were monounsaturated oleic acid (C18:1Δ^9^), polyunsaturated linoleic acid (C18:2Δ^9,12^) and saturated palmitic acid (C16:0), while minor were monounsaturated palmitoleic (C16:1Δ^9^), saturated stearic acid (C18:0) and polyunsaturated linolenic acid (C18:3Δ^6,9,12^) (with some exceptions in lipid classes, which will be explained later). Regarding available literature, Viljoen *et al.* (*38*) in *P. stipitis* grown on glucose/maltose as carbon source detected oleic, linoleic, palmitic and palmitoleic acids as the most abundant FAs. Grillitsch *et al.* (*39*) detected oleic, linoleic, α-linolenic acid (C18:3Δ^9,12,15^) and palmitic acids as the most abundant fatty acids in related species *P. pastoris* grown on glucose as a carbon source, while in the research of Kaneko *at al*. (*36*) two different species of *Pichia* genus (*P. membranaefaciens* and *P*. *farmosa*) differ in the relative ratio of the most abundant FAs. In *P. membranaefaciens* oleic acid prevailed (with 41%) followed by linoleic (23.6%), while in *P*. *farmosa* linoleic acid prevailed (46.4%) followed by palmitic (25.5%) and oleic (23.9%) acids. Unsaturated and C18 FAs were prevalent in both species, although the fractions differ in different species, which is in accordance with the results obtained in this research. In the fraction of total lipids, unsaturated fatty acids (UFA) and C18 FAs prevailed, making more than 80% in both tested strains (Table 2). Comparing the wild type with M12 strain, there was a statistically significant difference in the mass fraction of almost all identified FAs (except C16:0). M12 strain was characterised by similar mass fraction of oleic and linoleic acids, while in the wild type the mass fraction of linoleic acid was 1.8 times higher than that of oleic acid.

**Table 2:** Main fatty acids (FA, expressed as mass fraction of total identified fatty acids) of the total, neutral and polar lipids and free fatty acids in *Sheffersomyces stipitis* wild type and M12 yeast strain and the main features of the composition

Fatty acid	*w*(FA)/%							
Total lipids		Neutral lipids		Polar lipids		Free fatty acids	
Wild type	M12 strain	Wild type	M12 strain	Wild type	M12 strain	Wild type	M12 strain
C 15:0	n.d.	n.d.	2.6±0.6	1.9±0.6	n.d.	(3.1±1.0)*	4.9±0.5	(0.03±0.05)*
C 16:0	14.5±0.4	13.6±0.5	20.9±2.9	(16.0±0.6)*	10.8±0.3	(17.3±1.2)*	45.4±2.5	(22.0±1.4)*
C 16:1	3.0±0.3	(2.4±0.1)*	2.4±0.3	1.6±0.7	3.0±0.3	(2.3±0.3)*	6.5±0.4	(5.0±0.7)*
C 18:0	3.0±0.5	(5.4±0.2)*	5.0±0.4	(7.7±0.9)*	2.1±0.3	(4.4±0.5)*	9.2±1.4	(5.0±0.9)*
C 18:1	27.5±0.8	(38.4±1.1)*	26.6±2.2	(44.4±1.6)*	25.4±0.4	(31.4±0.7)*	30.5±3.5	(40.4±1.6)*
C 18:2	48.3±1.1	(37.0±1.1)*	38.6±2.6	(25.7±2.1)*	53.2±1.1	(32.1±1.7)*	2.1±0.5	(25.9±0.4)*
C 18:3	3.2±0.2	(2.8±0.1)*	1.9±0.6	(1.1±0.3)*	4.7±0.1	(2.1±0.3)*	0.1±0.1	(1.4±0.3)*
C 20:1	0.23±0.05	0.21±0.02	1.1±0.4	0.6±0.2	1.1±0.4	0.6±0.2	0.8±0.0	n.d.*
c 22:3	0.08±0.03	0.08±0.03	n.d.	(0.4±0.2)*	0.3±0.1	(3.2±0.9)*	n.d.	n.d.
c 22:4	0.10±0.02	0.09±0.02	0.7±0.1	0.6±0.2	0.4±0.1	(3.5±1.1)*	0.4±0.2	n.d.*
UFA	82.4±0.7	(81.0±0.3)*	71.3±4.0	74.5±1.7	88.1±0.7	(75.2±1.0)*	40.4±0.6	(72.7±0.8)*
mono-	30.7±0.3	(41.0±0.2)*	30.1±2.2	(46.6±1.6)*	29.5±0.2	(34.3±0.6)*	37.9±0.2	(45.4±0.6)*
poli-	51.7±0.9	(40.0±0.8)*	41.2±2.6	(27.8±2.1)*	58.6±1.0	(40.9±1.2)*	2.6±0.3	(27.3±1.2)*
C16	17.5±0.7	(16.00±0.5)*	23.3±0.7	(17.6±0.5)*	13.8±0.3	(19.6±1.6)*	51.9±0.2	(27.0±1.4)*
C18	82.0±0.6	(83.6±0.5)*	72.1±2.1	(78.9±0.6)*	85.4±0.6	(70.0±0.4)*	42.0±0.7	(72.7±1.3)*
SFA/UFA	0.21±0.01	(0.24±0.01)*	0.41±0.09	0.35±0.03	0.15±0.01	(0.33±0.02)*	1.47±0.03	(0.43±0.01)*
IU	1.38±0.02	(1.24±0.02)*	1.17±0.09	(1.06±0.01)*	1.52±0.01	(1.35±0.03)*	0.44±0.01	(1.02±0.03)*

Mean value±S.D. of at least three independent experiments performed in duplicate was calculated on the basis of peak areas; *significantly different from the wild type (Mann-Whitney *U* test, p<0.05), n.d.=not detected

The mass fraction of neutral lipid fraction (Table 2) is similar to total lipid mass fraction, but that of linoleic acid is 10% lower in both strains, while in M12 strain the content of oleic acid in neutral lipids is even higher than in total lipids (comprising 44% mass fraction).

The mass fraction of polar lipids is quite interesting – in M12 strain the mass fraction of palmitic, stearic and oleic acids was higher, the mass fraction of palmitoleic, linoleic and linolenic acids lower than in the wild type and C15:0 was also determined. In total, the mass fraction of saturated fatty acids (SFA) was 12% higher in M12 strain than in wild type. The content of C22:3 and C22:4 in M12 strain was also interesting – it was 8-10 times higher than in the wild type, each making more than 3% of mass fraction and it represents the highest measured content (comparing all tested fractions).

The composition of the FFA fraction in the wild type was completely different from all the other fractions. It was characterised by abundance of palmitic acid (making more than 45%) and the highest content of stearic acid (more than 9%), resulting in the 60% SFA, and the highest C_16_/C_18_ ratio compared to M12 strain, and all the other fractions. Regarding the FFA fraction in M12 strain, oleic and C18 FAs prevailed, making 40% and more than 72% of total mass fraction.

Linoleic and oleic acids had similar values in the TAG fraction of the wild type, while oleic acid significantly prevailed in M12 strain (Table 3). Comparing the content of SFA and UFA, and their ratio in M12 TAG and M12 neutral lipid fraction, they are almost equal. In the SE fraction of the wild type, oleic acid slightly prevailed, comprising 44%, UFAs made more than 90% of total FAs, and C_18_ more than 88%. In M12 strain, oleic acid also prevailed, but the content of linoleic acid was significantly lower, and that of palmitic acid higher than in wild type.

**Table 3:** Main fatty acids (FA, expressed as mass fraction of total identified fatty acids) of the triacylglycerols and sterol esters in *Sheffersomyces stipitis* wild type and M12 strain and the main features of the composition

Fatty acid	*w*(FA)/%			
Triacylglycerols		Sterol esters	
Wild type	M12 strain	Wild type	M12 strain
C 16:0	14.3±1.0	13.7±0.8	7.1±0.8	(11.4±1.1)*
C 16:1	2.2±0.2	(1.7±0.2)*	3.3±0.2	(2.70±0.05)*
C 18:0	8.0±0.6	(11.2±1.4)*	1.6±0.2	1.8±0.6
C 18:1	35.9±0.6	(46.1±1.5)*	44.0±1.7	45.9±0.8
C 18:2	36.6±0.9	(25.1±1.7)*	41.5±1.1	(35.6±1.1)*
C 18:3	1.8±0.1	(1.3±0.3)*	1.3±0.2	1.4±0.3
C 20:0	0.5±0.2	0.3±0.2	n.d.	n.d.
C 20:1	0.2±0.1	0.4±0.3	0.5±0.1	0.48±0.09

UFA	76.9±0.9	(74.6±0.7)*	90.6±1.1	(86.1±0.8)*
mono-	38.4±0.3	(48.2±0.7)*	47.8±1.3	49.1±1.6
poli-	38.5±0.9	(26.4±0.8)*	42.8±1.00	(37.0±2.1)*
C16	16.5±0.8	15.4±0.3	10.4±0.9	(14.1±1.1)*
C18	82.4±0.8	(83.7±0.5)*	(88.4±0.7)	(82.9±1.4)*
SFA/UFA	0.30±0.02	(0.34±0.01)*	0.10±0.01	(0.17±0.01)*
IU	1.18±0.02	(1.03±0.02)*	1.36±0.02	(1.25±0.01)*

Mean value±S.D. of at least three independent experiments performed in duplicate was calculated on the basis of peak areas; the results represent the mean value; *significantly different from the wild type (Mann-Whitney *U* test, p<0.05)

In all tested fractions, the content of UFA correlated well with the index of unsaturation (IU). Comparing the IU in the wild type and M12 strain, there was a statistically significant difference between these two strains in all tested fractions. Interestingly, in all fractions except in the FFA, the IU in M12 strain was lower than in the wild type, mainly because of lower value of linoleic acid and higher of oleic acid. The greatest difference in the IU was in the FFA fraction (0.44 in wild type and 1.02 in M12 strain). All other values (except 0.44) were greater than 1, and the highest was in PL of the wild type (1.51). It is known that UFAs play an essential role in the biophysical characteristics of cell membranes. They are also important for proper functioning of membrane-attached proteins. Cells maintain the proper fluidity of membrane lipids by altering the degree of unsaturation in their membranes. PL with high IU and UFA have a lower melting point and more flexibility than the phospholipids with saturated acyl chains and ordered membrane structure (*40*). FA desaturases that incorporate unsaturated bonds at defined positions in FAs are responsible for such adaptation. In *Pichia pastoris* Δ^12^ and Δ^15^-fatty acid desaturases are found (*21*, *22*). These two desaturases produce several polyunsaturated fatty acids (PUFA), including linoleic and linolenic acid (*21*). Prielhofer *et al.* (*20*) confimed the transcriptional induction of FAD12 gene and its increase in *Pichia sp*. grown on glycerol (Fig. 2). The presence of PUFA is very important, because they contribute to a more favourable membrane environment for efficient exocytotic/secretory processes (*39*).

In both tested *S. stipitis* strains the IU had the highest value in the PL fraction (Table 2). The value is higher in the wild type than in M12 PLs (1.52 *vs* 1.35), mainly because of higher content of palmitic acid and lower linoleic acid in M12 strain. Except in the difference in the IU and FA composition in the PL fraction, these two types of yeast also showed great difference in plasma membrane phospholipid composition (data not shown). Grillitsch *et al.* (*39*) dealt with phospholipid composition and explained in detail phospholipid composition of whole cells and plasma membrane of *P. pastoris* grown on different carbon sources. Briefly, asymmetrical arrangement in the two membrane leaflets is typical for plasma membrane, where phosphatidylcholine and sphingolipids are mostly found in the outer membrane leaflet, and negatively charged phospholipids (phosphatidylserine and phosphatidylinositol) in the inner leaflet. Sterols are also important structural components. Hydrophobic and stereochemically rigid by nature, they decrease the fluidity of the membranes. Although all these membrane lipids are in continuous turnover depending on biosynthetic, metabolic and degrading processes, the change in their content influences the function of this compartment. Besides many other functions, the plasma membrane helps in the import of components into the cell and the export of molecules into the surroundings. The organism we dealt with in this work also has very important functions along with SAM production, and we can assume that our tested strain adjusts itself for this important process by changing the phospholipid and sterol content and FA composition and by desaturation adjustments. This is even more emphasized in M12 strain as the content of ergosterol, which is usually a major sterol, is reduced (*8*). Therefore, in M12 strain, besides ergosterol and fecosterol, different sterols build membranes with different structure and hydrophobicity. This requires more PUFAs or different structural adjustments to maintain proper membrane fluidity.

Likewise, coordinated action of all metabolic pathways in M12 strain helps to achieve growth and intense SAM production. Fig. 2 shows a network of actual metabolic pathways constructed to offer insight into the lipid remodelling pathways in *S. stipitis* wild type and M12 strain during growth on glycerol and SAM production. The network was mainly constructed based on the data analysis given by Prielhofer *et al.* (*20*) for *P. pastoris* grown on excess of glycerol. The data from Klug (*15*), Tae Myeong *et al.* (*19*), Ivashov *et al.* (*13*, *18*), Wei *et al.* (*21*) and Zhang *et al.* (*22*) were also implemented in this network.

#### Network of actual biochemical pathways in *S. stipitis* wild type and M12 strain grown on glycerol

According to Klein *et al.* (*41*), two pathways for the dissimilation of glycerol in yeasts have been reported: the so called ’catabolic G3P pathway’ and ’the catabolic DHA pathway’. Yeast used in this study metabolises glycerol using the first one. l-glycerol 3-phosphate (G3P) is formed as the intermediate and a glycerol kinase (Gut1) and a FAD-dependent glycerol 3-phosphate dehydrogenase (Gut2) are the main enzymes. Phosphatidic acid (PA) could be synthesized from G3P and dihydroxy acetone phosphate (DHAP) produced in this step. PA is involved in further processes as a central precursor for TAG and phospholipid synthesis (*17*). According to the obtained results, it seems that all these synthetic processes were disregarded in M12 strain (the content of TAG and PL was significantly lower than in the wild type, Table 1). The obtained results are consistent with Prielhofer *et al.* (*20*), who showed the transcriptional repression of almost all transcripts needed in TAG and PL synthesis grown on glycerol (SLC1, CDS1, LRO1, DPP1 shown in Fig. 2).

Degradation of glycerol continues with lower glycolysis reactions, acetyl-CoA formation, tricarboxylic acid (TCA), glyoxylate cycle and pyruvate dehydrogenase (PDH) pathway. According to Prielhofer *et al.* (*19*) glycerol increases transcriptional level of many TCA cycle genes, isocitrate lyase (Icl1) and malate synthase (Dal7) involved in the glyoxylate cycle, phosphoenolpyruvate carboxykinase (Pck1), a key enzyme in gluconeogenesis that catalyses the formation of phosphoenolpyruvate from oxaloacetate, as well as mitochondrial aldehyde dehydrogenase (Ald4-1) and acetyl-coA synthetase (Acs1) that are part of PDH bypass (Fig. 2). The glyoxylate cycle is an additional pathway occurring in plants, fungi, bacteria and protists. The unique enzymes of this route are Icl1 that cleave isocitrate to glyoxylate and succinate, and Dal7 that converts glyoxylate and acetyl-CoA to malate. The end products of this bypass can be used, among other, for gluconeogenesis (*42*). In PDH bypass, pyruvate is converted to acetaldehyde by the enzyme pyruvate decarboxylase (Pdc1) and later into acetate by the enzyme Ald4. The acetate is then activated and converted to acetyl-CoA by the Acs1. The intermediate acetaldehyde is a branch point which could finally be converted to acetyl-CoA or ethanol (*43*). This bypass is probably an additional way how *S. stipitis* produces acetyl-CoA.

Considering the results published by Prielhofer *et al.* (*20*) of gene-specific response to glycerol as carbon source and comparing this with the results obtained in this work, it seems that active gluconeogenesis and glyoxylate cycle occur in both tested *S. stipites* strains.

Prielhofer *et al.* (*20*) also reported that while growing on glycerol, FA oxidation is induced by FA utilization genes (*e.g*. all genes involved in β-oxidation FAA2, FOX2, POT1, POX, *etc*. have approx. 2-fold higher transcript levels on glycerol than on excess of glucose). It seems that for both types of *S. stipitis* strains tested in this work, β-oxidation is a prominent pathway and the most important source for ATP, needed in the SAM production. Moreover, metabolome analysis provided by Hayakawa *et al.* (*44*) showed that the level of intracellular ATP was depleted in the methionine-supplemented medium. In order to ensure effective SAM production, the increase of ATP supply is crucial.

In wild type, during normal growth on glycerol, degradation of TAG is not favourable process. TAG is the major nonpolar lipid, synthesised with the main function to serve as a reservoir of energy and building blocks for membrane lipids. Synthesis and degradation of TAG are regulated mainly in response to the carbon source (*45*). Lower level of TAG obtained in this work in M12 strain than in wild type could be explained by its degradation (Fig. 2). It is known that TAGs are normally degraded in a cascade of hydrolytic reactions. They are hydrolysed to DAG and FA using Tgl3 and Tgl4; enzymes that are also a DAG lipase, and degrade DAG to MAG and FA. In the next step MAG is hydrolysed to glycerol and FA by the action of MAG lipase, Yju3 (*19*, *45*). The results obtained in this work show that M12 strain during the arduous period of SAM production, besides the above-mentioned ways, uses degradation of TAG to fulfil energy needs. Moreover, the accumulation of FFA in M12 is noticed, together with the absence of DAG and MAG fractions (in both types of tested yeasts). Significantly increased content of SE in M12 strain is also indirectly connected to this. SE, which are usually storage lipids (*17*, *45*), in *S. stipitis* M12 strain do not have the same function. It seems that they serve to tidy up the excess of sterols; the increase of the content of SE fraction and decrease of sterols content are actually adjustment of M12 strain to the growth conditions.

## CONCLUSIONS

A coordinated action of all metabolic pathways achieved high biomass yield and intensive *S*-adenosyl-l-methionine (SAM) production in *Sheffersomyces stipitis* M12 strain grown on glycerol with pulsed addition of methionine. By changing the content and composition of its lipids (mainly phospholipids, sterols and sterol esters) and with desaturation adjustments, our tested strain adjusts itself to fulfil increased energy needs for intensive metabolite production. This fundamental knowledge of lipid metabolic pathways will be a helpful tool in improving *S. stipitis* as an expression host and a model organism, opening new perspectives for applied research of this yeast strain.
